# Socioeconomic Inequity in Access to Medical and Long-Term Care Among Older People

**DOI:** 10.1186/s12939-024-02345-7

**Published:** 2025-01-23

**Authors:** Shohei Okamoto, Atsuhiro Yamada, Erika Kobayashi, Jersey Liang

**Affiliations:** 1 Research Team for Social Participation and Healthy Aging, Tokyo Metropolitan Institute for Geriatrics and Gerontology, 35-2 Sakae-cho, Itabashi-ku, Tokyo, 1730015 Japan; 2 Faculty of Economics, Keio University, Tokyo, Japan; 3 Department of Health Management and Policy, School of Public Health, University of Michigan, Michigan, USA; 4 Department of Healthcare Management and Healthy Aging Research Center, Chang Gung University, Taoyuan, Taiwan

## Abstract

**Background:**

Ensuring equitable access to medical and long-term care (LTC) is critical to enable older people to maintain their health and well-being even after they undergo a decline in their intrinsic capacity.

**Methods:**

We used data from five waves of the National Survey of the Japanese Elderly, conducted between 2002 and 2021, to assess gradients in access to medical care and LTC by income and education among Japanese individuals aged 60 years and above. Specifically, we assessed self-reported unmet needs for medical care and LTC, and public LTC use, and estimated the concentration indices (CI) to evaluate the degree of inequality and inequity. We standardised public LTC use by need and non-need variables. We analysed data derived from up to 1,775 person-wave observations from 1,370 individuals.

**Findings:**

The pooled incidence across waves of forgone medical care, self-reported unmet support for activities of daily living (ADL) or instrumental ADL (IADL), and those not certified for LTC services even with ADL or IADL limitations were 4.6%, 15.5%, and 62.5%, respectively. Public LTC use demonstrated pro-higher education and pro-rich distribution, whereas the gaps decreased for need-predicted use. Based on the CI estimates, no explicit inequality was found for forgone medical care. However, we observed inequity in standardised LTC use across education, indicating pro-higher education inequality, particularly among women and those aged ≥ 80 years.

**Conclusion:**

Improving the understanding of available resources and strengthening the functions of health centres and communities are required to detect the needs of citizens and facilitate their access to necessary care.

**Supplementary Information:**

The online version contains supplementary material available at 10.1186/s12939-024-02345-7.

## Background

Universal health coverage (UHC) means everyone, everywhere can access nationally determined sets of needed essential health services, including promotion, prevention, treatment, rehabilitation, and palliative care without incurring financial hardship [1]. Older people may require more care to maintain their functional ability (i.e. the interaction of intrinsic capacity with environmental characteristics), human dignity, and well-being when their intrinsic capacity (i.e. a combination of their physical and mental capacities) declines [2]. Ageing population is a global trend [3]; therefore, ensuring equitable access to necessary care, including long-term care (LTC) for older people, is essential to achieve progress toward UHC and attain the right to health.

LTC includes numerous services required by people dependent on assistance with the basic activities of daily living (ADL) [4]. Among the OECD countries, the total LTC expenditure per country’s gross domestic product ranges from 0.1 to 4.1%, in addition to differences in care providers (e.g. nursing homes, hospitals, home care, households, and social providers) [5]. To date, only six countries have adopted national social LTC insurance systems, namely the Netherlands, Israel, Germany, Luxembourg, the Republic of Korea, and Japan [6]. Japan is the first Asian country, which has adopted a public universal LTC insurance in 2000 [7]. It principally covers all citizens aged 40 years or older. To be eligible for public LTC benefits, people requiring care undergo an objective test to evaluate their level of ADL dependence and an assessment by their attending physician and are classified into independence, support-need-levels 1 to 2, and LTC need-levels 1 to 5. The types and amounts of available benefits are determined based on this classification. The users bear charges for LTC services at a 10% co-payment rate (or 20% or 30% depending on one’s income level). In addition, several financial protection policies are available to mitigate catastrophic out-of-pocket payments and the financial burden of insurance premiums for low-income individuals. Further details on LTC insurance in Japan have been described previously [7].

## Socioeconomic Inequity in Access to Care and Unmet LTC Needs

Despite evidence of socioeconomic inequity in access to healthcare [8–10], there is limited evidence of such inequity in LTC. Based on the Grossman model [11], socioeconomic inequity in access to health care can arise from various channels, such as disparities in health literacy, income, time preference, and available time for producing health. However, inequitable access to LTC can be driven by different channels from health care, including those due to substitution between formal and informal care. Those unable to access the necessary care experience unmet needs, which could lead to negative health outcomes [12, 13]; or ensuring access to LTC can have a protective effect for increased medical care expenditures, such as one for emergency care use [14]. Women, lower education, and lower economic status predict unmet needs for personal assistance in ADL among older adults [15, 16]. Inequity in LTC access exists in the Republic of Korea and the US, suggesting the importance of extending insurance coverage and subsidising low-income individuals [17–19]. Formal care among people without dementia is pro-rich, and poor people are more likely to experience unmet care needs in England [20]. Furthermore, findings from Spain and the Survey of Health, Aging, and Retirement in Europe suggest that formal services are concentrated among richer people, whereas poorer people tend to use intensive informal care [21, 22]. Studies in the Netherlands also find a pro-poor gradient in home care use [23, 24]. However, a Japanese study found that formal LTC use was generally equitable across income groups [25].

LTC services may still exhibit economic inequity despite the availability of universal insurance or benefits, as suggested by studies in the Republic of Korea and European countries [17, 18, 22]. Nevertheless, income inequity in LTC service use among those eligible for LTC benefits has not been observed in Japan [25], potentially owing to its low co-payment rate and financial protection policies. However, as mentioned before, socioeconomic inequity in care access can arise from financial and non-financial causes. Under the social insurance scheme, users need to contract with providers to purchase services at a (quasi) market, which would require a certain level of knowledge about LTC services and their own preferences in service use. Furthermore, in Japan, individuals are required to submit an application to be eligible for public LTC services, which may serve as a hindrance to accessing care for non-financial reasons. Therefore, we aimed to expand the literature in assessing socioeconomic inequity in LTC access using both financial and non-financial indicators under the public, universal LTC insurance system.

## Methods

### Data

Data were obtained from the National Survey of the Japanese Elderly [26], comprising Japanese adults aged ≥ 60 years. The survey was first conducted in 1987 and was followed up with participants every 3 to 6 years, adding new samples to complement sample size declines caused by deaths and a loss to follow-up. The sample was extracted from the Basic Resident Registration System using a stratified two-stage random sampling method based on a combination of regional blocks and population. The details of the survey are available on the project website [26]. From waves 1 to 10, 7,892 individuals responded in one or more waves. To evaluate access to LTC services, we analysed data collected from wave 6 (2002) to wave 10 (2021), following the introduction of public LTC insurance in 2000.

We restricted our analysis to participants who responded to interviews by themselves, excluding mail, proxy, and non-responders’ surveys because LTC-need-related information and self-reported unmet needs were only available for these people. Thus, we obtained 10,743 person-wave observations from 5,471 unique, non-institutionalised individuals from Wave 6 to Wave 10. We then focused on those with LTC needs as described below, leading to 1,975 person-wave observations by 1,512 individuals. Finally, we excluded those with missing information, resulting in the final sample size of at most 1,775 person-wave observations by 1,370 individuals. The detailed sample size calculation is provided in Appendix Figure A-1.

In this study, we evaluated inequality (i.e. differences across population groups) and inequity (i.e. need-adjusted disparities) in access to care in the following procedures.

### Access to Medical Care: Forgone Care

To assess inequality in medical care access, we used self-reported forgone medical care [10, 27]. In the survey, for the question, ‘During the past 3 months, how often did you reduce the dose of medication or did not see a physician even though it was necessary?’, each respondent selected one of the following options: (1) most times; (2) sometimes; (3) hardly/none; and (4) they did not need to consult a physician or consume medicine. Respondents who selected ‘most times’ or ‘sometimes’ were regarded as forgoing needed care. We excluded those who did not need to consult a physician or consume medicine to make individuals comparable regarding their health needs. To assess inequality in medical care access, we analysed the data obtained in waves 8, 9, and 10, because the questionnaire was administered only in these waves.

### Access to LTC

1. Self-reported unmet needs

Respondents with LTC needs were questioned if someone else (e.g. family members and LTC workers) had helped them with their activities within the past 3 months. They rated the frequency of receiving support as follows: (1) almost always; (2) sometimes; (3) occasionally; (4) never; and (5) did not need support. Respondents who selected ‘sometimes’, ‘occasionally’, or ‘never’ were categorised as having experienced an unmet need for LTC. This operational definition is reasonable because people who cannot obtain necessary care whenever they need them experience unmet need.

To define the LTC needs operationally, we used questionnaires on difficulties in performing basic and instrumental activities of daily living (ADL and IADL). Similar to the Katz index [28], ADL was measured using the following six items: bathing, dressing, feeding, transferring, outing, and toileting. IADL was measured using the following four items: shopping for personal items, using a telephone, riding the bus or subway alone, and performing light tasks around the house [29]. Each item was rated on a five-point Likert scale, including 0 (never difficult), 1 (moderately difficult), 2 (very difficult), 3 (extremely difficult), and 4 (unable to do at all). Respondents experiencing difficulties with at least one of the items (i.e. 1. moderately difficult to 4. unable to do it at all) were defined as having LTC needs.

(2) Utilisation-based measurement

Considering health disparities across socioeconomic statuses [30, 31], the care need differs across groups, leading to dissimilar levels of care utilisation. Thus, higher utilisation among the poor with worse health status does not imply pro-poor inequity in care access based on the vertical equity principle. Therefore, standardised utilisation must be used to assess inequity in access to care [32].

We standardised LTC use by the LTC need explained by demographics, health status, and morbidity status [32], formalised as a non-linear model with repeated measures (i.e. multilevel mixed-effects logistic regression) as follows:

$$\:{y}_{it}=\frac{\text{e}\text{x}\text{p}(\alpha\:+\sum\:_{j}{\beta\:}_{j}{x}_{jit}+\sum\:_{k}{\gamma\:}_{k}{z}_{kit}+{u}_{t})}{1+\text{e}\text{x}\text{p}(\alpha\:+\sum\:_{j}{\beta\:}_{j}{x}_{jit}+\sum\:_{k}{\gamma\:}_{k}{z}_{kit}+{u}_{t})}$$

where $\:{y}_{it}$ denotes a binary variable corresponding to 1 if respondent *i* in year *t* is considered eligible for public LTC services at any level, and 0 otherwise. The *j*th need variables ($\:x$) to predict LTC use and *k*th control variables ($\:z$) to avoid omitted-variables bias, with parameters $\:\beta\:$ and $\:\gamma\:$, are included. $\:\alpha\:$ is a constant, and $\:u$ is the random effects. Accepting that the variance of need-standardised use may depend on the formulation of the $\:z$ variables in the standardisation procedure, need-standardised LTC use ($\:{\widehat{y}}_{it}^{ST}$) is defined as:

$$\begin{aligned}{\widehat{y}}_{it}^{ST}&={y}_{it}-\frac{\text{exp}\left(\widehat{\alpha\:}+\sum\:_{j}\widehat{{\beta\:}_{j}}{x}_{jit}+\sum\:_{k}{\widehat{r}}_{k}{\stackrel{-}{z}}_{k}+{u}_{t}\right)}{1+\text{exp}\left(\widehat{\alpha\:}+\sum\:_{j}\widehat{{\beta\:}_{j}}{x}_{jit}+\sum\:_{k}{\widehat{r}}_{k}{\stackrel{-}{z}}_{k}+{u}_{t}\right)}\\&+\frac{1}{n}\sum\:_{i}\frac{\text{exp}\left(\widehat{\alpha\:}+\sum\:_{j}\widehat{{\beta\:}_{j}}{x}_{jit}+\sum\:_{k}{\widehat{r}}_{k}{\stackrel{-}{z}}_{k}+{u}_{t}\right)}{1+\text{exp}\left(\widehat{\alpha\:}+\sum\:_{j}\widehat{{\beta\:}_{j}}{x}_{jit}+\sum\:_{k}{\widehat{r}}_{k}{\stackrel{-}{z}}_{k}+{u}_{t}\right)}\end{aligned}$$

where $\:n$ denotes the sample size, and the $\:z$ variables are set to their means ($\:\stackrel{-}{z}$). The need variables directly influencing LTC needs are proxied by the demographic and morbidity characteristics [32], including the age, gender, self-rated health, the number of chronic conditions (ranging from 0 to 6), the degree of difficulty in performing each ADL (0–24) and IADL (0–16), and cognitive functioning (0–9). These need variables are considered to influence one’s LTC use due to their health needs. A previous study shows that individuals tend to use LTC services when they are older, female, and have more chronic conditions [33]. Moreover, ADL, IADL, and cognitive functioning are critical to evaluate one’s LTC needs because eligibility for LTC service utilisation in Japan is judged based on one’s levels in these areas [7].

The non-need control variables include the marital status, the number of co-resident members, residential area, the population size of the residential area, and year dummies. These non-need variables may affect the health status through social support and relationships [34]; however, they are expected to mainly affect formal and informal care availability, generating potential heterogeneity across group behaviour in the application and utilisation of formal LTC services. The need and non-need variables are defined in the Appendix Table A-2.

### Empirical Strategy

#### Concentration Index

To measure inequality and horizontal inequity in the access to medical and LTC services across socioeconomics groups, we estimated the concentration indices (CI) frequently used to evaluate health disparities [32]. The CI in each year is defined as twice the area between the concentration curve and the 45° line as follows [32]:

$$\:C=\frac{2cov({h}_{i},\:{R}_{i})}{\stackrel{-}{h}}=\:\frac{1}{n}\sum\:\left\{\frac{{h}_{i}}{\stackrel{-}{h}}(2{R}_{i}-1)\right\}$$

where $\:{h}_{i}$ denotes the health variable of interest to measure inequity, and $\:\stackrel{-}{h}$ is its mean. $\:{R}_{i}$ is the rank variable in which the health gradients are measured. For binary outcomes, the concentration index (W) was rescaled to consider its dependence on the variable means and to satisfy the mirror condition, i.e. the absolute value of a measured inequality is symmetric when computed over either attainments or shortfalls as follows [35]:

$$\:W=\:\frac{1}{n}\sum\:\left\{\frac{{(a}^{max}-{a}^{min}){a}_{i}}{\left({a}^{max}-\stackrel{-}{a}\right)\left(\stackrel{-}{a}-{a}^{min}\right)}\left(2{R}_{i}-1\right)\right\}$$

The CI is positive when access is more frequent among higher socioeconomic groups and negative if otherwise. We used the years of education and income as the rank variables to measure inequality and inequity. For income, we used the couple’s gross annual income equivalised by the marital status (i.e. divided by the square root of 2 if married) and residualised by employment status (See Appendix B). To calculate the descriptive differences in LTC utilisation across education and income groups, but not the CI, both indicators were divided into five categories. The category for education included lowest (< 6), lower-middle [6–9], middle [9–12], higher-middle [12–15], and higher (16+). Despite different pre- and post-World War II educational systems, this categorisation roughly corresponded to the distinctions between elementary, secondary, high school, and university or higher. Income was divided into 20th percentile groups.

Demographic factors, such as gender and age, can influence help-seeking behaviours [36]; thus, we evaluated inequities according to the gender and age. Considering that the average age of the sample was approximately 81 (standard deviation: 6.28), we categorised the participants into those aged < 80 years and ≥ 80 years. This categorisation is reasonable because people can become drastically dependent on ADL around the age of 80 years [37].

The abovementioned method measures inequity in each survey year; thus, it focuses on short-run inequity. However, it is equally important to analyse the dynamics of health inequity. For this, previous studies have attempted to measure long-run inequity in health and health care utilisation [38, 39]. In this study, we also evaluated long-run inequity in LTC use by estimating the CIs from within-individual means of standardised LTC use, education, and income. In doing so, three points should be noted. First, LTC use was comparable over the period because it was standardised in the same way across time. Second, socioeconomic indicators and their ranks were considered to be stable in older people, given that years of education rarely change and that respondents’ income was equivalised and residualised by their employment status. Third, with cross-sectional and longitudinal weights mentioned below, biases caused by the frequency of observed periods were partially adjusted.

We also complemented our analysis by multilevel mixed-effects linear regression to understand the association between the standardised LTC use, education, and income across the observation period.

To partially adjust for potential selection bias, we adopted two types of weights, namely, cross-sectional and longitudinal. Cross-sectional weights were estimated by logistic regression as probabilities of responding to baseline surveys predicted by the age, gender, the geographic area of residence, and the municipal/population category of a residential area. Longitudinal weights were estimated as response probabilities in each wave, estimated by the age, gender, employment status, marital status, education, self-rated health, the geographic area and population category of a residential area at baseline or the closest. These approaches were similar to multiple imputations based on random missing data [40, 41].

## Results

### Descriptive Statistics

Table 1 summarises the incidence of forgone medical care and unmet LTC needs in each wave. The average incidence for forgone medical care was 4.6% across the waves as follows: 4.5%, 6.0%, and 3.8% in 2012, 2017, and 2021, respectively. The average incidence of unmet need for ADL or IADL support was 41.6%, ranging from 37.9 to 44.6% from 2002 to 2021. Approximately, 62.0% of the participants with ADL or IADL limitations were not certified for LTC services (55.3–71.8%).

**Table 1:** Incidence of unmet need for medical and long-term care

		2002	2006	2012	2017	2021	Total
Forgone medical care	%			4.5%	6.0%	3.8%	4.6%
	Total, N			2,044	1,264	1,542	4,850
Unmet need for ADL or IADL support	%	44.6%	40.4%	41.4%	37.9%	41.9%	41.6%
	Total, N	442	385	471	320	157	1,775
Non-certified for LTC services even with ADL or IADL limitations	%	71.8%	60.2%	59.1%	55.3%	58.1%	62.0%
	Total, N	442	385	471	320	157	1,775

*Note*. These are weighted by both cross-sectional and longitudinal weights

Table 2 defines the pooled descriptive statistics between Wave 6 (2002) and Wave 10 (2021) for those with LTC needs. Of all participants, 38.0% were certified for public LTC use. The average age was approximately 80.8 years, and the majority of participants were women (72.6%).

**Table 2:** Pooled descriptive statistics, Wave 6 (2002) to Wave 10 (2021)

Stats	% or mean	Standard deviation		%
Certified for long-term care use	38.0%		Residential area (Geographic region of Japan)	
Standardised long-term care use	0.38	0.44	Hokkaido	3.6%
Years of education	9.28	2.58	Tohoku	10.1%
Couple’s income (10,000JPY)	175.21	152.43	Kanto	18.8%
Age	80.80	6.28	Hokuriku	7.1%
Women	72.6%		Tozan	6.9%
Employment status: Working	5.1%		Tokai	8.0%
SRH: Very good	11.5%		Kinki	13.2%
SRH: Good	39.8%		Chugoku	9.7%
SRH: Fair	35.6%		Shikoku	4.3%
SRH: Bad	11.1%		Kyushu	18.3%
SRH: Very bad	2.0%		Municipal/population category: Government-designated	13.0%
N of chronic conditions	2.02	0.99	Population size: 200 K+	20.0%
ADL score (0–24)	2.77	3.87	Population size: 100–200 K	16.4%
IADL score (0–16)	4.79	4.22	Population size: < 100 K	28.1%
Memory test (0–9)	1.89	1.71	Population size: Town and villages	22.5%
N of household members	1.94	1.69		
Marital status: Single	55.1%			

*Note*: SRH stands for self-rated health; ADL stands for activities of daily living; IADL stands for instrumental activities of daily living; government-designated cities have populations greater than 500,000 and have been designated by the Cabinet of Japan; these descriptive statistics are calculated for 1,775 respondents with long-term care needs (i.e. ADL or IADL limitations), weighted by both cross-sectional and longitudinal weights; couples’ income is equivalised by the marital status and residualised by employment status, and the sample size is 1,375; individuals whose marital status is single include those divorced and widowed

Appendix Table A-2 presents the regression results to predict LTC use by need and non-need variables. Both needs (e.g. chronic conditions and ADL and IADL limitations) and non-needs (e.g. the number of co-resident members) were associated with the certification for public LTC use.

Table 3 summarises the mean probabilities of non-standardised, need-predicted, and need-standardised LTC use by education and income, pooling the data between waves 6 and 10. Remarkably, the actual distribution of public LTC was pro-higher-education and pro-rich, whereas the gaps decreased for need-predicted use. Thus, the participants with lower education and income used public LTC services less than expected by 3–7% points, whereas their counterparts with middle and higher levels used services more than expected by 3–26% points. Despite standardisation, higher education and income groups used more services than the lowest groups.

**Table 3:** Non-standardised and standardised long-term care use by education and income: Mean probabilities, pooled between Wave 6 (2002) and Wave 10 (2021)

		Actual	Need-predicted	Difference	Standardised
Education	Lowest: <6	0.30	0.37	-0.07	0.31
	Lower middle: 6–9	0.37	0.39	-0.03	0.35
	Middle: 9–12	0.46	0.38	0.08	0.45
	Higher middle: 12–15	0.42	0.39	0.03	0.40
	Higher: 16+	0.70	0.44	0.26	0.64
Income (Quintile)	Poorest 20%	0.34	0.37	-0.03	0.34
	2nd poorest 20%	0.35	0.38	-0.02	0.35
	Middle	0.42	0.38	0.04	0.42
	2nd richest 20%	0.49	0.38	0.10	0.48
	Richest 20%	0.42	0.37	0.05	0.42

*Note*: Income refers to couples’ income, which is equivalised by the marital status and residualised by employment status

### Socioeconomic Inequality and Inequity in Access to Medical Care and LTC

**Fig. 1:**
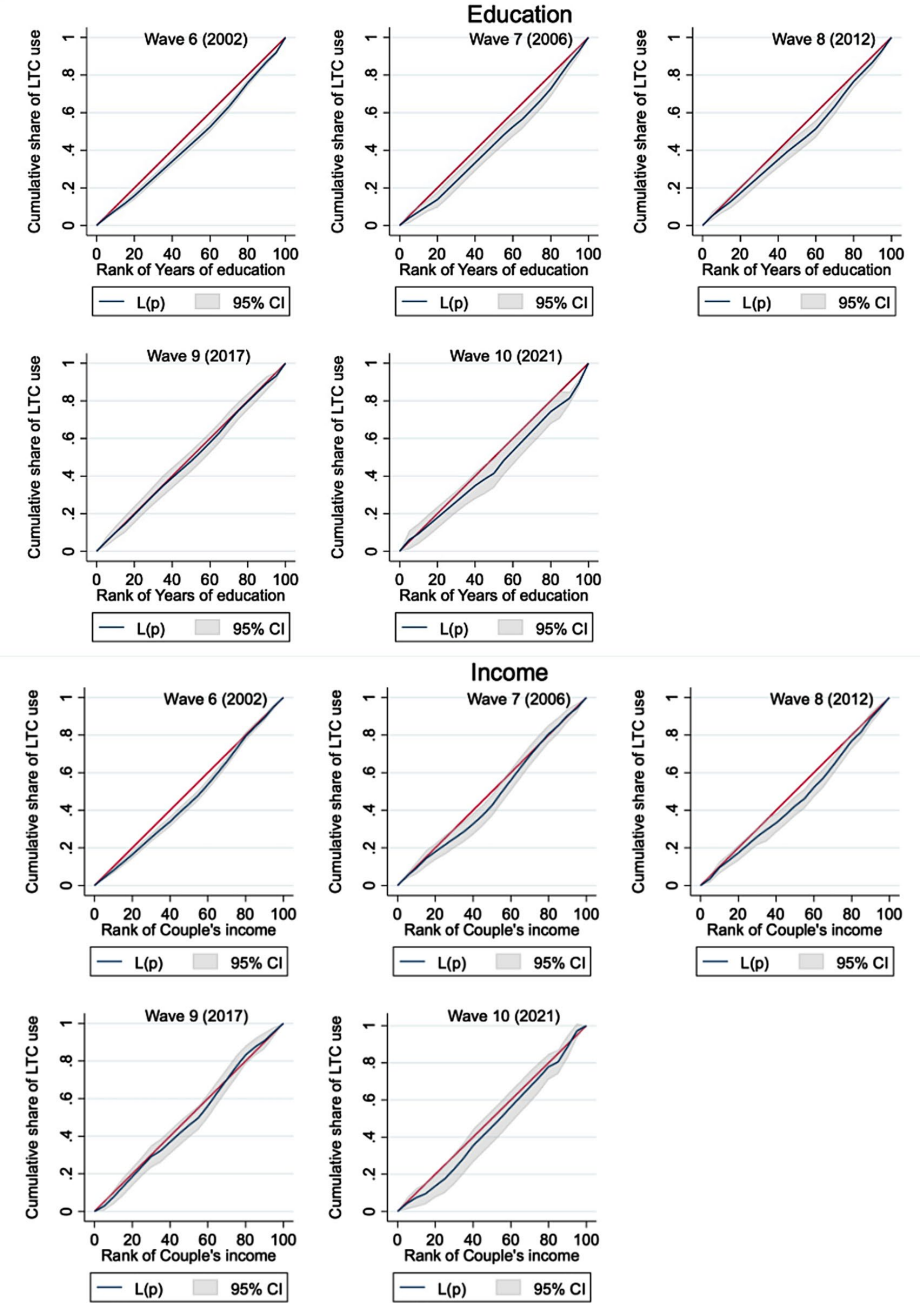
Concentration curves on standardised long-term care use by education and income in each wave. *Note*: The 95% confidence interval (CI; grey area) is calculated based on standard errors (SE) adjusted for clusters for each respondent; estimates are weighted by both cross-sectional and longitudinal weights. L(p) is the concentration curve for need-standardised long-term care (LTC) use. Couple’s income is equivalised by the marital status and residualised by employment status

Table 4 summarises CIs for medical care and LTC use by education and income in each wave. Figure 1 presents visualised horizontal inequities in LTC use by education and income. We observed no explicit inequality for forgone medical care. However, inequity was observed in LTC use across education in 2006 and 2012, indicating pro-higher-education inequality: CIs with standard errors were 0.12 (0.03) for 2006 and 0.09 (0.03) for 2012. Meanwhile, the inequity was less obvious for income, showing that concentration curves crossed 45-degree lines in most waves and pro-rich inequality was evident only in 2012. For non-need-standardised self-reported unmet needs for LTC support, both education and income inequality were evident in 2002.

**Table 4:** Concentration indices for medical and long-term care use in each wave

			2002	2006	2012	2017	2021
Forgone medical care	Education	Concentration index			-0.02	0.11	-0.02
		SE			0.07	0.07	0.08
		P-value			0.73	0.11	0.80
		N			2,044	1,264	1,542
	Income	Concentration index			-0.09	-0.00	0.04
		SE			0.08	0.08	0.09
		P-value			0.26	0.99	0.63
		N			1,744	1,103	1,224
Unmet need for long-term care	Education	Concentration index	-0.13	0.03	-0.00	-0.04	0.05
		SE	0.06	0.06	0.06	0.07	0.09
		P-value	< 0.05	0.60	0.99	0.55	0.58
		N	442	385	471	320	157
	Income	Concentration index	-0.14	-0.16	-0.07	-0.03	0.07
		SE	0.06	0.07	0.07	0.08	0.12
		P-value	< 0.05	< 0.05	0.30	0.71	0.54
		N	376	300	381	256	118
Non-standardised long-term care use	Education	Concentration index	0.11	0.22	0.12	-0.00	0.16
		SE	0.06	0.06	0.06	0.07	0.10
		P-value	0.08	< 0.01	< 0.05	0.96	0.09
		N	442	385	471	320	157
	Income	Concentration index	0.07	0.08	0.14	0.07	0.08
		SE	0.07	0.07	0.07	0.08	0.11
		P-value	0.34	0.25	< 0.05	0.42	0.49
		N	376	300	381	256	118
Standardised long-term care use	Education	Concentration index	0.07	0.12	0.09	-0.00	0.08
		SE	0.04	0.03	0.03	0.04	0.05
		P-value	0.05	< 0.01	< 0.01	0.94	0.13
		N	442	385	471	320	157
	Income	Concentration index	0.04	0.05	0.08	0.03	0.06
		SE	0.04	0.04	0.04	0.04	0.06
		P-value	0.32	0.17	< 0.05	0.43	0.33
		N	376	300	381	256	118

*Note*: Standard errors (SE) are adjusted for clusters in each respondent; Wagstaff indices are presented; and estimates are weighted by both cross-sectional and longitudinal weights. Income refers to couples’ income, which is equivalised by the marital status and residualised by employment status

### Heterogeneity by Gender and Age

Standardised LTC use was pro-higher-education and pro-rich for both men and women (Appendix Table A-3). CI-based inequities in each wave were particularly evident among women, corresponding to the results of our primary analysis (Appendix Table A-4). Regarding the heterogeneity by age, we observed similar pro-higher education, particularly among those aged 80 years (Appendices A-5 and A-6).

### Long-Run Inequity

**Fig. 2:**
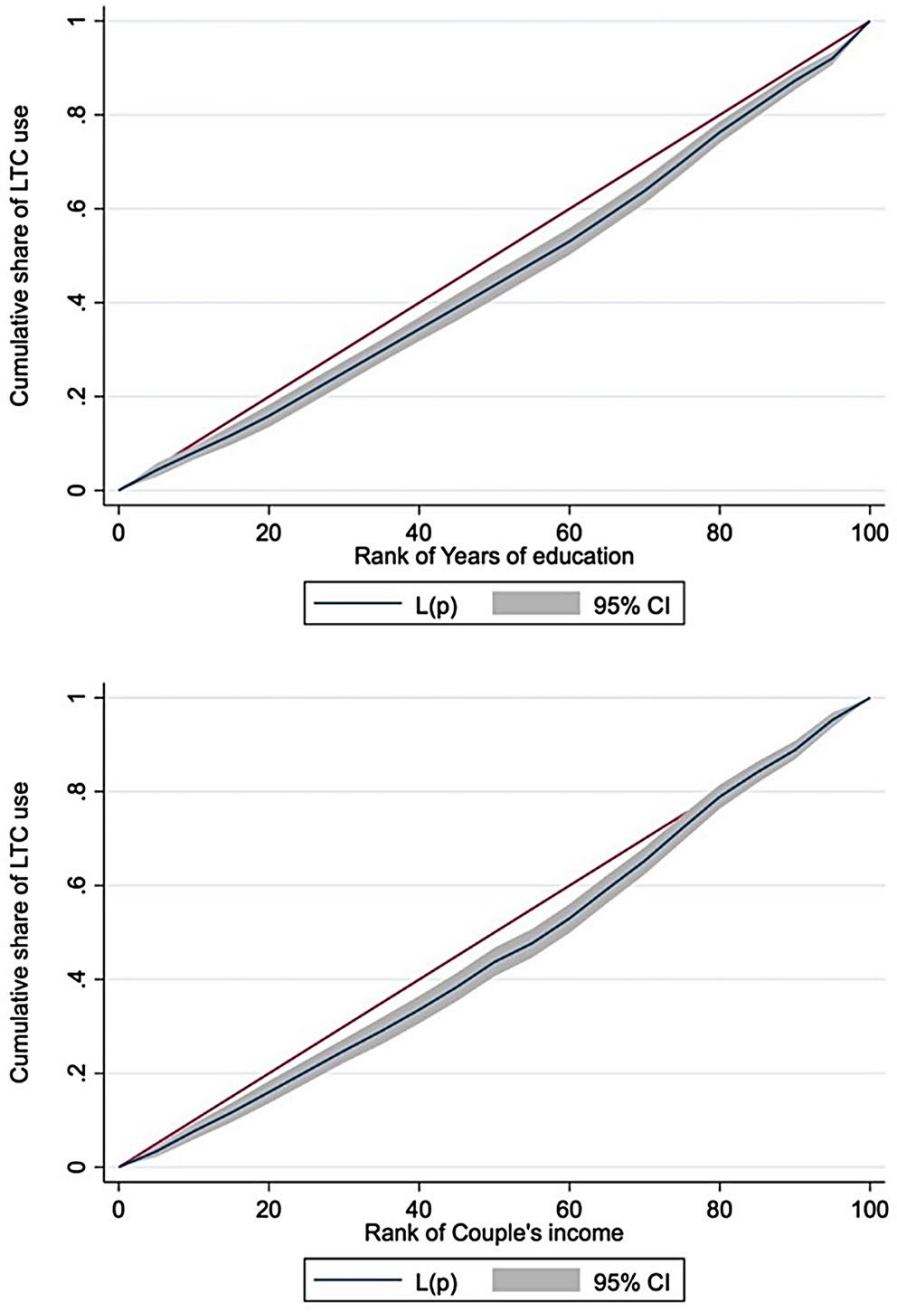
Concentration curves on standardised long-term care use by education and income, long-term trend. *Note*: The 95% confidence interval (CI; grey area) is calculated based on standard errors (SE) adjusted for clusters for each respondent; estimates are weighted by both cross-sectional and longitudinal weights. Couple’s income is equivalised by the marital status and residualised by employment status. L(p) is the concentration curve for need-standardised long-term care (LTC) use

Table 5; Fig. 2 present long-run inequity in LTC use by education and income. Over the period of maximum 19 years between 2002 and 2021, our analysis clearly shows educational inequity in LTC use among all respondents as well as men and women. CIs with standard errors were 0.08 (0.02) for all respondents, 0.07 (0.04) for men, and 0.10 (0.02) for women, revealing pro-higher-education inequity in all groups. Inequity was also evident for income, showing pro-rich inequity in LTC use among all respondents: The CI and standard error was 0.07 (0.02). By sub-group analysis, pro-rich inequity was evident only among women, with the CI of 0.10 and standard error of 0.02.

**Table 5:** Concentration indices for long-term care use, long-term trend

	Socioeconomic indicator	Statistics	Total	Men	Women
Standardised long-term care use	Education	Concentration index	0.08	0.07	0.10
		SE	0.02	0.04	0.02
		P-value	< 0.01	< 0.05	< 0.01
		N	1,370	431	939
	Income	Concentration index	0.07	0.01	0.10
		SE	0.02	0.04	0.02
		P-value	< 0.01	0.78	< 0.01
		N	1,155	383	772

Income refers to couples’ income, which is equivalised by the marital status and residualised by employment status

As a result of the complementary analysis using multilevel mixed-effects linear regression, we observed that lower educational attainment was associated with lower LTC use while higher educational attainment was associated with higher LTC use (Appendix Table A-7). However, the association was not evident for income. This finding was consistent with the results from other analyses in this study.

## Discussion

This study aimed to assess unmet need and socioeconomic inequity in medical care and LTC access. We obtained three main findings. First, 37.9–44.6% of the respondents experienced unmet needs for LTC, reporting that they received insufficient support for ADL and IADL. Relatedly, 55.3–71.8% were not certified for LTC service use even with ADL or IADL limitations. Second, socioeconomic inequity in LTC access was observed, showing pro-higher-education inequity and, to some extent, pro-rich inequity. Pro-higher-education inequity was evident even in the long-term analysis. Third, pro-higher-educational inequity was especially evident among women and the oldest-old population, despite being descriptively similar among their male and younger (i.e. 60–80 years) counterparts.

Even though the country contexts are not the same, our findings are consistent with previous studies in that demographic and socioeconomic factors are linked to unmet need for LTC [15–24]. In contrast to previous findings from Japan [25], these studies identified the economic determinants of LTC use and suggested the importance of financial protection. Our study found educational inequity in LTC access and indicated that merely providing financial protection is not sufficient to ensure equitable access to LTC. Unmet need and inequity in care access are caused by multiple factors, including issues related to availability, affordability, accessibility, and acceptability [42].

Japan has the highest UHC achievement [43], providing coverage for a wide range of health services and financial protection policies with public, universal health and LTC insurance; nonetheless, challenges exist with certain proportions of people experiencing unmet medical and LTC needs. People with higher education may be better at accessing the necessary services, considering health literacy is related to education [44] and predicts a lack of understanding and use of health services [45]. For medical care, older people might not experience unmet needs frequently due to the absence of gatekeeping and low copayment rates.

Educational inequity in LTC use was more pronounced among women and the old. This may be explained by different help-seeking behaviours by men and women despite similar health needs. Women are more likely to seek and use care than men [46] and older people on average are expected to have greater needs due to age-related changes in their physical and mental conditions; therefore, disparities could be more apparent when a specific group forgoes it.

Our findings generate policy implications for enhancing access to care. First, barriers to accessing LTC or relevant services may exist because people need to make an application by themselves. In Japan’s policies for care for older people, community comprehensive support centres located in each municipality play an important role in care management and facilitating the use of necessary care. Nevertheless, reaching out to these centres still requires care seeking from people with some difficulties themselves or their family members. For some older people, a decline in intrinsic capacity and care needs may be detected by annual national health check-ups or regular consultations with their attending physicians when they have chronic conditions. However, poor health is associated with social isolation [47]; therefore, people with health issues may be unable to access the necessary care because of physical barriers (e.g. the lack of transportation) or health-literacy-related issues (e.g. the lack of knowledge about how to seek care). Therefore, it is necessary to enhance access through any of these channels, such as education, to improve an understanding of available resources, strengthening the functions of health centres and communities to detect the needs of citizens, and facilitating their access to necessary care.

This study had four limitations. First, the sample size was small because we focused only on individuals with LTC needs. Therefore, we may not obtain significant results for some analyses or may be unable to generalise our findings. Second, our measure of LTC certification was self-reported and did not include information on the number of services used. Further studies should address these limitations by using extensive administrative data. Third, we could not distinguish between unmet needs for formal and informal LTC because of data restrictions. Depending on the policy context in each country, the availability and service coverage of formal long-term care services largely vary. Therefore, assessing the unmet needs for both formal and informal care comprehensively is required to design the service coverage of formal care. Fourth, there were non-responders, particularly in Wave 10. Thus, we may have missed vulnerable populations during the state of emergency in large cities, by which we cannot conclude that inequity was absent during the pandemic.

## Conclusions

In conclusion, approximately 42% of individuals with LTC needs experienced unmet need for ADL or IADL support, and approximately 62% were not certified for public LTC use even with ADL or IADL limitations. Inequity in LTC access was driven mainly by education rather than income. Even if the services are universally available at an affordable cost, financial and non-financial supports are indispensable to ensuring all people, especially the marginalised and vulnerable groups, can access care whenever and wherever they need them.

## Electronic supplementary material

Below is the link to the electronic supplementary material.

Supplementary Material 1

## Data Availability

Data up to wave 8 (2012) are currently available from the Social Science Japan Data Archive of the University of Tokyo. Recent waves will also be made available in due course.
