# Supplementary material for: Socioeconomic Inequity in Access to Medical and Long-Term Care Among Older People
Source: Int J Equity Health. 2025 Jan 23;24:28. doi: 10.1186/s12939-024-02345-7 (PMC11756124; doi:10.1186/s12939-024-02345-7)
Supplement: Supplementary file 1 — Supplementary Material 1 [file 12939_2024_2345_MOESM1_ESM.docx]

**Supplementary material for**

**Socioeconomic inequity in access to care among older people in Japan**

**Contents**

**Appendix A: Tables and Figures**

- Appendix Table A-1. A list of need and non-need variables
- Appendix Table A-2. Regression results: Prediction of long-term care use by need and non-need variables
- Appendix Table A-3. Gender-specific non-standardised and standardised long-term care use by education and income: Mean probabilities Pooled between Wave 6 (2002) and Wave 10 (2021)
- Appendix Table A-4. Concentration indices for long-term care use by gender
- Appendix Table A-5. Age-specific non-standardised and standardised long-term care use by education and income: Mean probabilities Pooled between Wave 6 (2002) and Wave 10 (2021)
- Appendix Table A-6. Concentration indices for long-term care use by age
- Appendix Table A-7. Multilevel mixed-effects linear regression result
- Figure A-1. Sample size calculation
- Figure A-2. Concentration curves on standardised long-term care use by non-residualised income

**Appendix B: Income Residualisation**

**Appendix A**

Appendix Table A-1. A list of need and non-need variables

| Classification | Variable | Description |
| --- | --- | --- |
| Need | Age | Respondent’s age |
|  | Gender | Respondent’s gender |
|  | Self-rated health | 1: Very bad, 2: Bad, 3: Fair, 4: Good, 5: Very good |
|  | Chronic conditions | The number of chronic conditions respondents suffer from, including chronic respiratory diseases, hypertension, heart diseases, diabetes, stroke, and cancer (0–6) |
|  | Activities of daily living (ADL) | A sum of the magnitudes of difficulties in performing bathing, dressing, feeding, transferring, outing, and toileting, ranging from 1: Never difficult to 5: Unable to do at all (0–24) |
|  | Instrumental activities of daily living (IADL) | A sum of the magnitudes of difficulties in shopping for personal items, using a telephone, riding the bus or subway alone, and performing light tasks around the house, ranging from 1: Never difficult to 5: Unable to do at all (0–16) |
|  | Cognitive functioning | The number of misreporting of cognitive functioning assessments comprising respondent’s home address, interview date, interview day, mother’s maiden name, name of the current prime minister, name of the previous prime minister, a simple calculation, respondent’s birthday, and respondent’s age (0–9) |
| Non-need | Marital status | 1 if single (including unmarried, divorced, and widowed); 0 otherwise |
|  | Co-residence | The number of family members living together with a respondent |
|  | Residential area (Geographic region of Japan) | Hokkaido, Tohoku, Kanto, Hokuriku, Tozan, Tokai, Kinki, Chugoku, Shikoku, and Kyushu |
|  | Municipal/population category  of residential area | Government-designated, 200K+, 100K-200K, <100K, and Towns and villages |
|  | Year | Wave 6 (2002), Wave 7 (2006), Wave 8 (2012), Wave 9 (2017), Wave 10 (2021) |

Appendix Table A-2. Regression results: Prediction of long-term care use by need and non-need variables

| Variables | beta (SE) | Variables | beta (SE) |
| --- | --- | --- | --- |
| Age: 60–64 | -1.44 (1.27) | Geographic region: Hokkaido | -0.06 (0.32) |
| Age: 65–69 | -0.35 (0.39) | Geographic region: Tohoku | 0.13 (0.25) |
| Age: 70–74 | -0.41 (0.26) | Geographic region: Kanto | Ref. |
| Age: 75–79 | -0.30 (0.19) | Geographic region: Hokuriku | -0.67* (0.30) |
| Age: 80–84 | -0.13 (0.14) | Geographic region: Tozan | 0.00 (0.30) |
| Age: 85+ | Ref. | Geographic region: Tokai | 0.04 (0.27) |
| Women | -0.05 (0.16) | Geographic region: Kinki | 0.20 (0.23) |
| SRH: Very bad | Ref. | Geographic region: Chugoku | -0.09 (0.24) |
| SRH: Bad | 0.20 (0.21) | Geographic region: Shikoku | 0.41 (0.35) |
| SRH: Fair | -0.32 (0.22) | Geographic region: Kyushu | -0.09 (0.22) |
| SRH: Good | -0.53# (0.27) | Municipal/population category: Government-designated | Ref. |
| SRH: Very good | -0.56 (0.43) | Municipal/population category: 200K+ | -0.20 (0.22) |
| ln(Chronic conditions) | 0.40** (0.14) | Municipal/population category: 100–200K | -0.27 (0.23) |
| ln(ADL difficulties) | 0.44** (0.07) | Municipal/population category: <100K | -0.66** (0.22) |
| ln(IADL difficulties) | 0.49** (0.09) | Municipal/population category: Towns and villages | -0.34 (0.24) |
| ln(Cognitive functioning) | -0.10 (0.08) | Wave 6 (2002) | Ref. |
| Marital status: Single | 0.27# (0.16) | Wave 7 (2006) | 0.46* (0.17) |
| ln(N of co-resident members) | -0.32** (0.09) | Wave 8 (2012) | 0.46** (0.17) |
| Variance(_cons[id]) | 0.00 (0.00) | Wave 9 (2016) | 0.64** (0.20) |
| Constant | -1.95** (0.46) | Wave 10 (2021) | 0.67** (0.23) |
| Observations | | 1,855 | |
| Number of unique individuals | | 1,431 | |

Note: Robust standard errors (SE) are in parentheses; estimates are weighted by both cross-sectional and longitudinal weights; ADL stands for activities of daily living; SRH stands for self-rated health; ** p<0.01, * p<0.05, # p<0.1

Table A-3. Gender-specific non-standardised and standardised long-term care use by education and income: Mean probabilities, Pooled between Wave 6 (2002) and Wave 10 (2021)

| Gender |  |  | Actual | Need-predicted | Difference | Standardised |
| --- | --- | --- | --- | --- | --- | --- |
| Men | Education | Lowest: <6 | 0.38 | 0.42 | -0.04 | 0.33 |
|  |  | Lower middle: 6–9 | 0.34 | 0.40 | -0.06 | 0.32 |
|  |  | Middle: 9–12 | 0.42 | 0.41 | 0.01 | 0.39 |
|  |  | Higher middle: 12–15 | 0.38 | 0.39 | -0.02 | 0.36 |
|  |  | Higher: 16+ | 0.70 | 0.45 | 0.25 | 0.62 |
|  | Income  (Quintile) | Poorest 20% | 0.36 | 0.39 | -0.02 | 0.35 |
|  |  | 2nd poorest 20% | 0.30 | 0.39 | -0.10 | 0.28 |
|  |  | Middle | 0.43 | 0.39 | 0.04 | 0.41 |
|  |  | 2nd richest 20% | 0.41 | 0.41 | -0.01 | 0.37 |
|  |  | Richest 20% | 0.45 | 0.40 | 0.05 | 0.42 |
| Women | Education | Lowest: <6 | 0.29 | 0.36 | -0.07 | 0.30 |
|  |  | Lower middle: 6–9 | 0.38 | 0.39 | -0.01 | 0.36 |
|  |  | Middle: 9–12 | 0.47 | 0.37 | 0.10 | 0.47 |
|  |  | Higher middle: 12–15 | 0.45 | 0.38 | 0.07 | 0.45 |
|  |  | Higher: 16+ | 0.69 | 0.38 | 0.32 | 0.69 |
|  | Income  (Quintile) | Poorest 20% | 0.33 | 0.37 | -0.04 | 0.34 |
|  |  | 2nd poorest 20% | 0.40 | 0.36 | 0.04 | 0.41 |
|  |  | Middle | 0.42 | 0.37 | 0.05 | 0.42 |
|  |  | 2nd richest 20% | 0.53 | 0.37 | 0.15 | 0.53 |
|  |  | Richest 20% | 0.38 | 0.33 | 0.05 | 0.43 |

Note: Income refers to couples’ income, which is equivalised by the marital status and residualised by employment status.

Table A-4. Concentration indices for long-term care use by gender

| Gender | | | 2002 | 2006 | 2012 | 2017 | 2021 |
| --- | --- | --- | --- | --- | --- | --- | --- |
| Men | Education | Concentration index | 0.02 | 0.10 | 0.06 | -0.02 | 0.15 |
|  |  | SE | 0.10 | 0.06 | 0.06 | 0.07 | 0.08 |
|  |  | P-value | 0.85 | 0.10 | 0.36 | 0.79 | 0.05 |
|  |  | N | 130 | 103 | 135 | 114 | 57 |
|  | Income | Concentration index | -0.11 | 0.12 | 0.10 | 0.00 | -0.06 |
|  |  | SE | 0.09 | 0.07 | 0.06 | 0.07 | 0.08 |
|  |  | P-value | 0.21 | 0.09 | 0.12 | 0.94 | 0.49 |
|  |  | N | 120 | 79 | 119 | 99 | 46 |
| Women | Education | Concentration index | 0.10 | 0.14 | 0.11 | 0.02 | 0.02 |
|  |  | SE | 0.04 | 0.04 | 0.04 | 0.05 | 0.07 |
|  |  | P-value | <0.01 | <0.01 | <0.01 | 0.64 | 0.76 |
|  |  | N | 312 | 282 | 336 | 206 | 100 |
|  | Income | Concentration index | 0.11 | 0.03 | 0.08 | 0.06 | 0.11 |
|  |  | SE | 0.04 | 0.04 | 0.05 | 0.05 | 0.08 |
|  |  | P-value | <0.01 | 0.42 | 0.07 | 0.31 | 0.18 |
|  |  | N | 256 | 221 | 262 | 157 | 72 |

Note: Standard errors (SE) are adjusted for clusters in each respondent; Wagstaff indices are presented; and estimates are weighted by both cross-sectional and longitudinal weights; Income refers to couples’ income, which is equivalised by the marital status and residualised by employment status.

Table A-5. Age-specific non-standardised and standardised long-term care use by education and income: Mean probabilities, Pooled between Wave 6 (2002) and Wave 10 (2021)

| Age |  |  | Actual | Need-predicted | Difference | Standardised |
| --- | --- | --- | --- | --- | --- | --- |
| 60–79 | Education | Lowest: <6 | 0.27 | 0.31 | -0.04 | 0.33 |
|  |  | Lower middle: 6–9 | 0.32 | 0.37 | -0.05 | 0.32 |
|  |  | Middle: 9–12 | 0.36 | 0.34 | 0.02 | 0.40 |
|  |  | Higher middle: 12–15 | 0.32 | 0.40 | -0.07 | 0.30 |
|  |  | Higher: 16+ | 0.59 | 0.39 | 0.20 | 0.57 |
|  | Income  (Quintile) | Poorest 20% | 0.33 | 0.35 | -0.02 | 0.36 |
|  |  | 2nd poorest 20% | 0.37 | 0.38 | -0.01 | 0.36 |
|  |  | Middle | 0.46 | 0.33 | 0.12 | 0.50 |
|  |  | 2nd richest 20% | 0.30 | 0.34 | -0.04 | 0.33 |
|  |  | Richest 20% | 0.32 | 0.32 | 0.00 | 0.37 |
| 80+ | Education | Lowest: <6 | 0.31 | 0.39 | -0.07 | 0.30 |
|  |  | Lower middle: 6–9 | 0.39 | 0.40 | -0.02 | 0.36 |
|  |  | Middle: 9–12 | 0.50 | 0.40 | 0.10 | 0.47 |
|  |  | Higher middle: 12–15 | 0.47 | 0.38 | 0.09 | 0.46 |
|  |  | Higher: 16+ | 0.78 | 0.48 | 0.30 | 0.67 |
|  | Income  (Quintile) | Poorest 20% | 0.34 | 0.38 | -0.04 | 0.33 |
|  |  | 2nd poorest 20% | 0.13 | 0.29 | -0.16 | 0.21 |
|  |  | Middle | 0.41 | 0.39 | 0.02 | 0.40 |
|  |  | 2nd richest 20% | 0.53 | 0.40 | 0.13 | 0.51 |
|  |  | Richest 20% | 0.47 | 0.39 | 0.08 | 0.45 |

Note: Income refers to couples’ income, which is equivalised by the marital status and residualised by employment status.

Appendix Table A-6. Concentration indices for long-term care use by age

| Age | | | 2002 | 2006 | 2012 | 2017 | 2021 |
| --- | --- | --- | --- | --- | --- | --- | --- |
| 60–79 | Education | Concentration index | 0.01 | 0.15 | 0.02 | -0.13 | 0.08 |
|  |  | SE | 0.05 | 0.06 | 0.07 | 0.18 | 0.07 |
|  |  | P-value | 0.76 | <0.05 | 0.75 | 0.48 | 0.29 |
|  |  | N | 207 | 118 | 102 | 33 | 58 |
|  | Income | Concentration index | 0.02 | 0.00 | 0.02 | 0.02 | 0.04 |
|  |  | SE | 0.05 | 0.06 | 0.08 | 0.16 | 0.09 |
|  |  | P-value | 0.65 | 0.98 | 0.78 | 0.89 | 0.65 |
|  |  | N | 184 | 98 | 80 | 28 | 43 |
| 80+ | Education | Concentration index | 0.13 | 0.12 | 0.13 | 0.03 | 0.06 |
|  |  | SE | 0.05 | 0.04 | 0.04 | 0.04 | 0.08 |
|  |  | P-value | <0.05 | <0.01 | <0.01 | 0.37 | 0.43 |
|  |  | N | 235 | 267 | 369 | 287 | 99 |
|  | Income | Concentration index | 0.07 | 0.09 | 0.11 | 0.04 | 0.09 |
|  |  | SE | 0.07 | 0.05 | 0.04 | 0.04 | 0.07 |
|  |  | P-value | 0.35 | 0.07 | <0.01 | 0.37 | 0.19 |
|  |  | N | 192 | 202 | 301 | 228 | 75 |

Note: Standard errors (SE) are adjusted for clusters in each respondent; Wagstaff indices are presented; and estimates are weighted by both cross-sectional and longitudinal weights; Income refers to couples’ income, which is equivalised by the marital status and residualised by employment status.

Appendix Table A-7. Multilevel mixed-effects linear regression result

| Outcome: Standardised long-term care use | | Total | Men | Women |
| --- | --- | --- | --- | --- |
| Education | Lowest: <6 | -0.12* (0.05) | 0.16 (0.12) | -0.17** (0.05) |
|  | Lower middle: 6–9 | -0.08* (0.04) | -0.03 (0.07) | -0.09* (0.05) |
|  | Middle: 9–12 |  |  |  |
|  | Higher middle: 12–15 | 0.04 (0.10) | 0.08 (0.12) | -0.00 (0.13) |
|  | Higher: 16+ | 0.26** (0.10) | 0.28** (0.10) | 0.51** (0.14) |
| Income  (Quintile) | Poorest 20% | -0.04 (0.05) | 0.01 (0.09) | -0.04 (0.05) |
|  | 2nd poorest 20% | -0.01 (0.06) | -0.06 (0.09) | 0.06 (0.08) |
|  | Middle |  |  |  |
|  | 2nd richest 20% | 0.08 (0.05) | 0.14 (0.11) | 0.09 (0.06) |
|  | Richest 20% | -0.06 (0.05) | -0.03 (0.07) | -0.00 (0.07) |
| Sample size | | 1,374 | 442 | 932 |

Note: Values presented here are coefficients with standard errors in parentheses; Controlled for age (each 5-year group), self-rated health, ln(the number of chronic conditions), ln(ADL score), ln(IADL score), ln(memory test), residential area, and municipal/population category; Three-level model with random intercepts by individuals and survey waves for survey waves nested within individuals; Income refers to couples’ income, which is equivalised by the marital status and residualised by employment status;.Full table is available upon request.

Figure A-1. Sample size calculation

Selected individuals, excluding those confirmed to have died before the surveys

| Total (Person-wave)  17,349 | | | | |  |
| --- | --- | --- | --- | --- | --- |
| Wave 6  (2002) | Wave 7  (2006) | Wave 8  (2012) | Wave 9  (2017) | Wave 10  (2021) |  |
| 3,877 | 3,263 | 4,364 | 2,257 | 3,588 |  |
| Valid responses | | | | |  |
| Total (person-wave)  10,735 | | | | |  |
| Wave 6  (2002) | Wave 7  (2006) | Wave 8  (2012) | Wave 9  (2017) | Wave 10  (2021) |  |
| 2,823 | 2,095 | 2,532 | 1,488 | 1,797 |  |
|  |  |  |  |  |  |
| Restricted to Those with LTC needs | | |  |  |  |
| Total (person-wave)  1,975 | | | | |  |
| Wave 6  (2002) | Wave 7  (2006) | Wave 8  (2012) | Wave 9  (2017) | Wave 10  (2021) |  |
| 490 | 430 | 517 | 345 | 193 |  |
|  |  |  |  |  |  |
| Final sample size: Excluded those with missing information used in this study | | | | |  |
| Total (person-wave)  1,775 | | | | |  |
| Wave 6  (2002) | Wave 7  (2006) | Wave 8  (2012) | Wave 9  (2017) | Wave 10  (2021) |  |
| 442 | 385 | 471 | 320 | 157 |  |

Figure A-2. Concentration curves on standardised long-term care use by non-residualised income


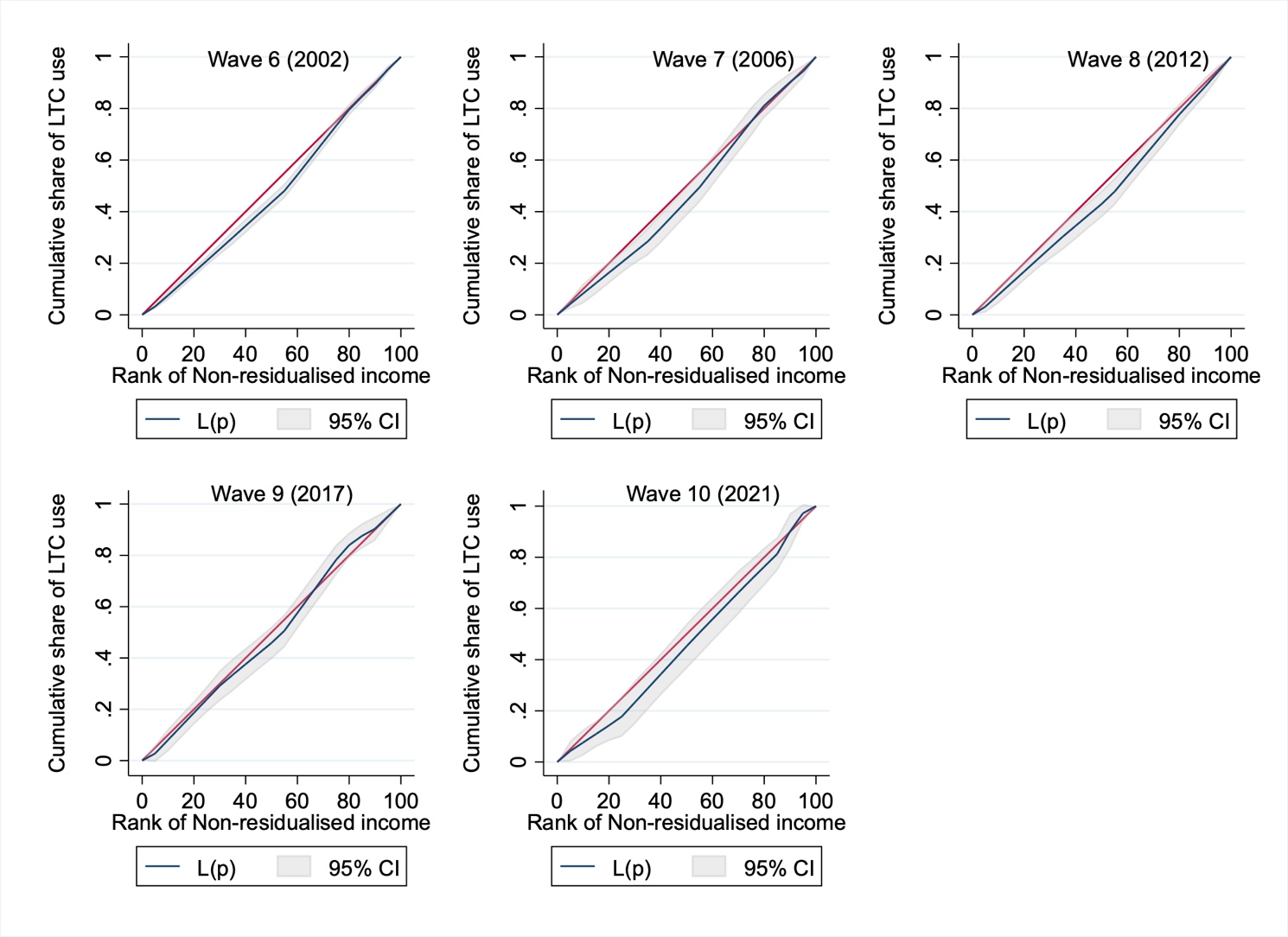


Note: The 95% confidence interval (CI) is calculated based on standard errors (SE) adjusted for clusters for each respondent; estimates are weighted by both cross-sectional and longitudinal weights. The sample sizes in each wave were 376, 300, 382, 256, and 118, respectively, and L(p) is the concentration curve for long-term care (LTC).

**Appendix B: Income Residualisation**

Employed and unemployed individuals are non-comparable because of large differences: theoretically, people decumulate their savings to smooth their consumption levels, particularly after retirement (i.e. life-cycle hypothesis) (1); Thus, a low income does not always suggest being deprived among older people. Instead of using limited information about savings and consumption from the National Survey of the Japanese Elderly, we residualised the income to partially adjust for the differences between workers and non-workers by fixed-effects ordinary least squares as follows:

$$e_{i, t}= Y_{i, t}- \tilde{Y}_{i, t}$$

$${ln(Y}_{i, t})=c+\delta_{i}{Work}_{i, t}+\theta_{i}\pi_{i, t}+\mu_{i}+ \in_{it}$$

where $e_{i, t}$ is the residualised income of individual i in year t, defined as the predicted income ($\tilde{Y}_{i, t}$) subtracted from the actual income ($Y_{i, t}$). The income is predicted by the employment status (${Work}_{i, t}$; =1 if in paid work; =0 if otherwise) and other independent variables ($\pi_{i, t}$), including the age, sex, marital status, residential area, municipal or population category of residential area, year-fixed effects, and individual fixed effects ($\mu_{i}$). $c$ is a constant, $\epsilon$ is the stochastic disturbance, and $\delta$and $\theta$ are the estimated coefficients of the independent variables. This formalisation enabled controlling for potential imbalances in the income arising from the employment status, demographic factors, price differences across areas and years, and time-invariant individual heterogeneity (Appendix Table B-1).

Appendix Table B-1. Regression result: Prediction of couple’s income

| Variables | beta (SE) |
| --- | --- |
| Age: 60–64 | Ref. |
| Age: 65–69 | -0.02 (0.02) |
| Age: 70–74 | -0.01 (0.03) |
| Age: 75–79 | -0.04 (0.05) |
| Age: 80–84 | -0.04 (0.05) |
| Age: 85+ | -0.05 (0.08) |
| Employment status: Currently working | 0.20** (0.02) |
| Marital status: Single | -0.06* (0.02) |
| Individual FE | Yes |
| Residential area FE | Yes |
| City-scale FE | Yes |
| Year FE | Yes |
| Constant | 5.13** |
|  | (0.03) |
| Observations | 17,031 |
| Number of unique individuals | 4,586 |

Note: Robust standard errors (SE) are in parentheses; estimates are weighted by both cross-sectional and longitudinal weights; FE denotes fixed effects; ** p<0.01, * p<0.05, # p<0.1

**References for Appendices**

1. Ando A, Modigliani F. The "Life Cycle" Hypothesis of Saving: Aggregate Implications and Tests. The American Economic Review. 1963;53(1):55-84.
